# Recombinant Production of a Novel Fusion Protein: Listeriolysin O Fragment Fused to S1 Subunit of Pertussis Toxin

**DOI:** 10.29252/ibj.25.1.33

**Published:** 2020-02-10

**Authors:** Hossein Forghani, Mahin Jamshidi Makiani, Hossein Zarei Jaliani, Mina Boustanshenas, Seyed Mohsen Zahraei

**Affiliations:** 1 Antimicrobial Resistance Research Center, Institute of Immunology and Infectious Diseases, Iran University of Medical Sciences, Tehran, Iran;; 2 Infectious and Tropical Diseases Research Center, Hormozgan Health Institute, Hormozgan University of Medical Sciences, Bandar Abbas, Iran;; 3 Department of Medical Biotechnology, School of Medicine, Shahid Sadoughi University of Medical Sciences, Yazd, Iran;; 4 Infectious Disease Center for Communicable Disease Control, Ministry of Health and Medical Education, Iran

**Keywords:** Adjuvant, Cloning, Fusion protein, Pertussis toxin

## Abstract

**Background:**

Some resources have suggested that genetically inactivated PTs bear a more protective effect than chemically inactivated products. This study aimed to produce new version of PT, by cloning an inactive PTS1 in a fusion form with N-terminal half of the LLO pore-forming toxin.

**Methods:**

Deposited pdb structure file of the PT was used to model an extra disulfide bond. Codon-optimized ORF of the PTS1 was used to make recombinant constructs of PTS1 and LLO-PTS1 in the pPSG-IBA35 vector. The recombinant PTS1 and LLO-PTS1 proteins were expressed in *BL21 DE3* and *SHuffle T7* strains of *E. coli* and purified by affinity chromatography. Cytotoxic effects of the recombinant proteins were examined in the MCF-7 cell line.

**Results:**

The purity of the products proved to be more than 85%, and the efficiency of the disulfide bond formation in *SHuffle T7* strain was higher than *BL21 DE3* strain. No cytotoxicity of the recombinant proteins was observed in MCF-7 cells. Soluble recombinant PTS1 and LLO-PTS1 proteins were produced in *SHuffle T7* strain of *E. coli *with high efficiency of disulfide bonds formation.

**Conclusion:**

The LLO-PTS1 with corrected disulfide bonds was successfully expressed in *E. coli* S*Huffle* *T7* strain. Due to the safety for human cells, this chimeric molecule can be an option to prevent pertussis disease if its immunostimulatory effects would be confirmed in the future.

## INTRODUCTION

Pertussis is a highly contagious respiratory illness caused by *Bordetella pertussis* bacteria^[^^1^^]^. This disease has been controlled utilizing two types of vaccines: the wP and aP ones^[^^2^^]^. The aP vaccines are subunit vaccines that contain pure and inactive components of B. pertussis cells^[^^3^^,^^4^^]^, including detoxified pertussis toxin, filamentous hemagglutinin, pertactin, fimbrial antigens and so on. However, the protectivity of the aP vaccines has not been adequate so that several outbreaks has been reported from different countries since 2012^[^^5^^,^^6^^]^. The causes of the weakness of aP vaccines are yet unclear because it is not known whether the re-emergence of pertussis is due to the vaccine waning immunity or to the fundamental differences in the nature of the immune response caused by aP vaccines compared with the wP vaccines or natural infection^[^^7^^]^. After the presentation of toxin-mediated theory about pertussis disease by Margaret Pittman in 1978^[^^8^^]^*, i*t was suggested that a suitable *inactivated* and immunogenic *PT* is necessary and sufficient for a pertussis vaccine^[^^9^^]^*. *Moreover, a former study reported that fimbria, similar to pertactin, is unnecessary for vaccine-induced immuno-stimulation^[^^10^^]^*. *PT is composed of two subunits: A subunit with ADP-ribosyltransferase activity (S1 subunit), which is the main antigen for protection, and B oligomer, which is responsible for the toxin attachment to the target cells and is an important protective antigen in all available pertussis vaccines^[^^11^^]^*. *

Currently, the toxoid components in aP vaccines are chemically inactivated by aldehyde agents. However, more immunogenic PTs are inactivated genetically and are expected to have a longer protective effect than current products, because of the better preservation of the antigenic epitopes[9]. The use of adjuvants in vaccine formulation is also important. Researchers believe that adjuvants can play a critical role in improving immunogenicity in next-generation aP vaccines^[^^12^^,^^13^^]^*. *LLO molecule has been the subject of many research studies, and its role as an adjuvant against several infection and cancer models has been clearly illustrated. In fact, it has been identified that LLO molecule can act as an adjuvant either in chimeric form or as a separately expressed molecule^[^^14^^-^^18^^]^*. *

In this study, a new mutant of PTS1 carrying an extra disulfide bond was designed. This chimeric molecule (LLO-PTS1) was then expressed in *BL21 DE3* and *SHuffle *T7 strains of *E. coli* and characterized further*. *

## MATERIALS AND METHODS

The *E. coli* DH5α and BL21 DE3 strains were procured from the Pasteur Institute of Iran (Tehran). SHuffle®T7 competent cells were prepared from New England Biolabs Inc., USA. DpnI restriction enzyme was obtained from Thermo Fisher Scientific (USA), and other chemicals were provided by molecular biology grade providers (Sigma and Merck Co., Germany). 

***Disulfide bond prediction***

1PRT.pdb file, MODIP software of the National Centre for Biological Sciences Integrated Web ServerV1.0.1^[^^19^^]^, and Disulfide By Design software version 2.12^[^^20^^]^ were used to model, generate files and analyze the new desired disulfide bond in the structure of the pertussis S1 enzymatic subunit. Three-dimensional graphics were generated using YASARA molecular graphics suite^[^^21^^]^*.*

**Gene synthesis**

The mutant gene of *PTS1 *was codon-optimized and chemically synthesized by Pishgaman Gene Transfer Co. (Iran). The synthetic fragment was received in the pUC57 cloning vector. Mutant PTS1 used in the present study had the following mutations: R9K, R13L, and E129G.

**Recombinant construct preparation**

**Fig. 1:**
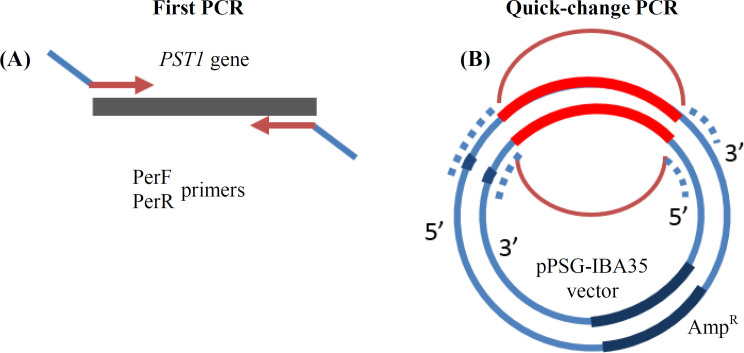
Design of the mutant PTS1 cloning method. (A) For introducing the *PTS1* gene into pPSG-IBA35 expression vector, two fragments of the multiple cloning site (blue extension) of the vector were added to the inserted gene by a simple PCR. (B) The product of the first PCR was used as a megaprimer in a second quick-change PCR to replace a fragment of the vector by the *PTS1* gene

**Fig. 2:**
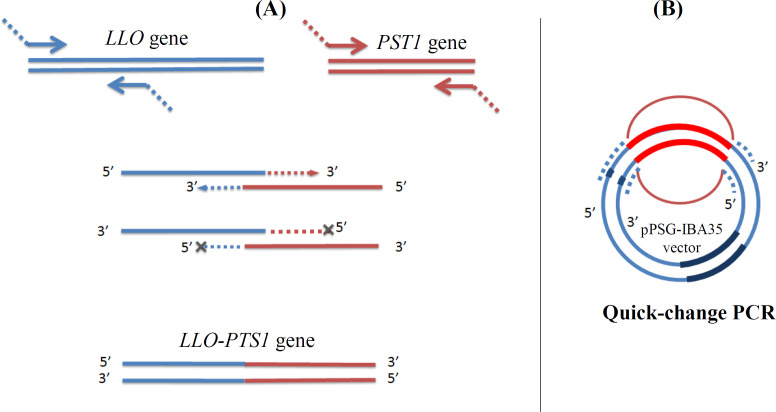
Design of the fusion LLO-PTS1 cloning method. (A) Two gene fragments were amplified in separate PCRs and ligated together in a SOEing PCR protocol. (B) For introducing the *LLO-PTS1* gene into pPSG-IBA35 expression vector, the product of the SOEing PCR was used as a megaprimer in a second quick-change PCR to replace a fragment of the vector by the *LLO-PTS1* gene

A set of primers (PerF and PerR) was applied for the amplification of the PTS1 gene. Each primer had a 5’ arm that was complementary to each side of the vector backbone, as illustrated in Figure 1. The PCR amplification of the PTS1 fragment was carried out using the above primers to result in PTS1 contain complementary ends to the pPSG-IBA35 expression vector backbone. The PCR product with the size of about 750 bp was used in a quick-change PCR to synthesize the whole vector with PTS1 gene inserted between the two arms. Another set of primers employed for the amplification of LLO gene (codon 26-266, which is N-terminally his-tagged) had already been sub-cloned in pPSG-IBA35. Codons of the LLO toxin, used in the present study, were codons 22-266. This part of the LLO comprised mostly of the D1 and D2 domains of the protein. Forward primer was complementary to T7 promoter. Reverse primer was hybridized to the last codons of N-terminal half of the LLO gene (up to codon 266) and had a 5' arm, which was complementary to the 5'-end of the PTS1 gene with a Gly2SerGly2 linker (Fig. 2). In addition, the PTS1 gene was amplified with a set of primers, at the forward with a 5' arm complementary to the last codons of LLO and the linker, and at the reverse with a 5' arm hybridizing to the vector backbone. The PCR amplification of LLO and PTS1 genes with these primers were performed to synthesize two gene fragments. The fragments were fused together using a SOEing PCR protocol, thus leading to a PCR product of about 1500 bp size harboring arms complementary to the pPSG-IBA35 vector backbone. This PCR product was used in a quick-change PCR to synthesize the whole vector with LLO-PTS1 fusion gene inserted between the two arms. DpnI-treated PCR products were transformed into chemically competent DH5α bacterial cells using the heat shock method. A single colony from each transformation reaction was cultured in 5-ml LB broth containing 50 μg.mL^-1^ of ampicillin at 37°C for 16 hours. The recombinant plasmids were extracted by column method (QIAprep Spin Miniprep kit, Qiagen^TM^, Germany), and the sequence of the construct was confirmed by DNA sequencing.

**Recombinant protein expression and purification**

Twenty nanograms of the purified plasmids were used to transform *BL21 DE3* and *SHuffle T7* competent cells by the heat shock method. Multiple colonies of *BL21 DE3* and *SHuffle* *T7 *transformants were cultured in a LB medium containing 50 μg.mL^-1^ of ampicillin at 37 °C and 28 °C, respectively, induced by the addition of 0.1 mM of IPTG when OD^600nm^ reached approximately 0.7. Expression of the recombinant proteins PTS1 and LLO-PTS1 was checked in a number of colonies. The fresh aliquots of recombinant protein expressing clones were used for the inoculation of another 100 mL of the LB medium. Clones were grown until the OD^600nm^ of 0.7, and the expression was induced by adding 0.1 mM of IPTG. After an overnight induction, the bacteria were harvested by centrifugation at 4500 ×g at room temperature for 10 min. Bacterial pellet was bead-beaten 15 times for 20 seconds with a bench-top vortex with maximum shaking speed (2000 rpm) with 30-second intervals of cooling on ice. Lysis buffer contained Tris (100 mM; pH 8.0), NaCl (300 mM), and glycerol (10%). The soluble fraction was centrifuged, and the supernatant was loaded onto a column filled with Ni- NTA agarose resin (Qiagen) to purify the His-tagged recombinant protein. Equilibration and washing buffers of the chromatography were the same as the lysis buffer. Elution buffer was also the same as the lysis buffer, except for the 300 mM of imidazole. Eluted fractions were analyzed by 12.5% SDS-PAGE for the presence of the recombinant protein, and fractions containing the recombinant protein were dialyzed against the phosphate buffer (pH 8.0) containing 50 mM of NaCl.

**Disulfide bond determination**

Free SH groups of recombinant proteins purified from *BL21 DE3* and *SHuffle T7* *E. coli *strains were examined in a denatured condition (pre-incubating proteins with 6 M of GuHCl for 15 min) using Ellman’s reagent. Concentrations of recombinant protein and 5,5′-Dithiobis(2-nitrobenzoic acid) were 2 μM and 1 mM in a cuvette of 300-μl volume, respectively. The absorbance of samples at 412 nm was measured, and the number of free SH groups was calculated for the recombinant PTS1 and LLO-PTS1 proteins purified from *BL21 DE3* and *SHuffle T7* strains, as stated before^[^^22^^]^.

**Cell culture and MTT assay**

Human cell line MCF-7 was purchased from the Pasteur Institute of Iran (Tehran). Cells were cultured and maintained in RPMI-1640 medium supplemented with 10% FBS, streptomycin and penicillin (1%), pH 7.4, in a humidified atmosphere of 95% air plus 5% CO_2_ at 37 °C. The anti-proliferative activity of PTS1 and LLO-PTS1 proteins in different concentrations was measured by the MTT assay. Cell viability of each well was calculated as [A^570^ of treated cells/A^570^ of control untreated cells] × 100%.

## Results

***Disulfide bond prediction***

**Fig. 3:**
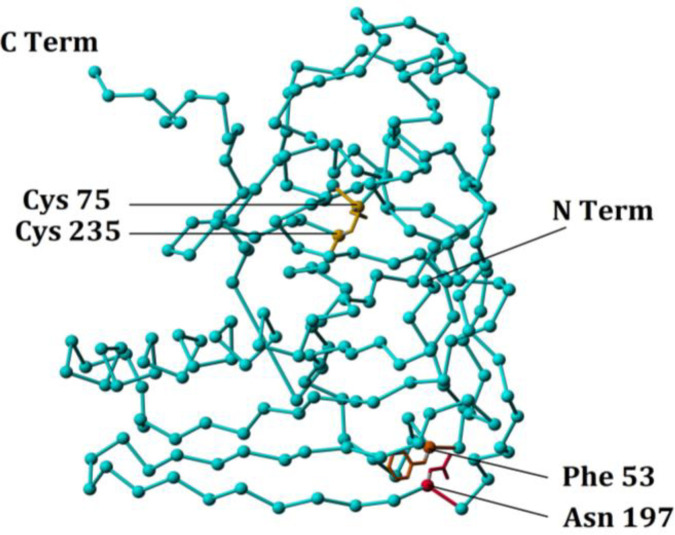
Modeling of the new disulfide bond in the mutant *PTS1* gene. Two amino acids, Phe53 and Asn197, were chosen to convert to Cys to form a new disulfide bond. Native disulfide bond of the PTS1 molecule is also shown. The numbering of the amino acids was performed concerning the Met of the signal sequence as the first amino acid

 The structure of the PTS1 was extracted from 1PRT.pdb file deposited in the PDB database. After an energy minimization step, using the MODIP program and Disulfide By Design, the best disulfide bridges were analyzed, and one of which was selected to introduce into the PTS1 gene. The distance of the disulfide bond partners from each other in the linear sequence of the protein, ΔG, and dihidral bond is among the parameters concerned with the selection of the best disulfide bond. The potential disulfide bond selected in the present study was Phe53-Asn197 with the numbering of the amino acids based on the first methionine of the PTS1 signal sequence (Fig. 3).

**Fusion gene and recombinant construct preparation**

**Fig. 4:**
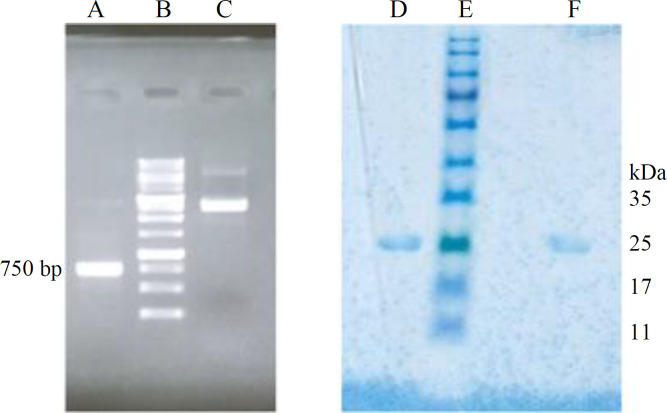
Quick-change PCR and purity of the mutant PTS1 protein. Quick-change PCR of the mutant *PTS1* gene was performed using the whole gene as megaprimer to introduce the gene into the expression vector pPSG-IBA35. Lane A, *PTS1* gene; lane B, 1 kb DNA size marker; lane C, quick-change PCR product; lane D, mutant PTS1 protein purified from *BL21 DE3* strain; lane E, pre-stained protein size marker; lane F, mutant PTS1 protein purified from *SHuffle* *T7* strain

**Fig. 5:**
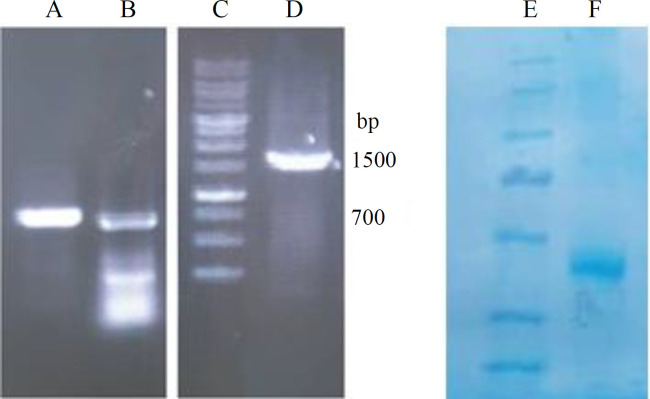
The SOEing PCR and purity of the LLO-PTS1 protein. SOEing PCR of the mutant *PTS1* gene and *LLO *gene fragment was performed to produce the fusion gene for using as a megaprimer to produce the expression construct of LLO-PTS1 in pPSG-IBA35 vector. Lanes A and B, PCR product of the amplification of the *PTS1* and *LLO* genes; lane C, 1 kb DNA size marker; lane D, SOEing PCR product; lane E, pre-stained protein size marker; lane F, mutant PTS1 protein purified from *SHuffle T7* strain

Designed *PTS1* gene was chemically synthesized and delivered in the pUC57 cloning vector. PCR of the gene using primers harbouring 5’ arms complementary to the *LLO* gene fragment and the pPSG-IBA35 vector backbone was carried out. In addition, the amplification of the coding sequence of the first 241 amino acids of LLO toxin was performed with designed primers. Quick-change PCR with amplified *PTS1* gene was performed, lanes A and C in Figure 4, to produce recombinant construct pPSG-PTS1. Moreover, the SOEing PCR led to the the megaprimer of about 1500 bp, as illustrated in Figure 5 (lane D). Quick-change PCR using this megaprimer was conducted to produce the recombinant construct pPSG-LLO-PTS1. 

**Expression and purification**

Recombinant N-terminal His-tagged PTS1 protein and LLO-PTS1 fusion protein were produced in *BL21 DE3* and *SHuffle T7* strains of *E. coli* in parallel using 0.1 mM concentration of IPTG, as an inducer. The purity and size of the recombinant proteins produced in *BL21 DE3* and *SHuffle T7* strains were the same (lanes D and F; Fig. 4). Yield of the total purified protein was 23.2 mg.L^-1^ and 13.1 mg.L^-1^ for the PTS1 protein and 19.4 and 17.4 mg.L^-1^ for the LLO-PTS1 fusion protein in the two strains, respectively, after the overnight induction of the recombinant protein expression. 

**Disulfide bond determination of the recombinant proteins**

The SH groups titration in denaturing condition indicated that the disulfide bonds of the recombinant mutant PTS1 protein purified from *SHuffle T7* strain have been formed with high efficiency. The ratio of the free SH groups for each molecule was calculated, which proved to be 0.21 and less than 0.05 for proteins

purified from *BL21 DE3* and *SHuffle T7* strains, respectively. Hence, the efficiency of disulfide bond formation in the two proteins was approximately 80% and over 94%, respectively. The same results were procured for the LLO-PTS1 protein. 

**Cell culture and MTT assay**

**Fig. 6:**
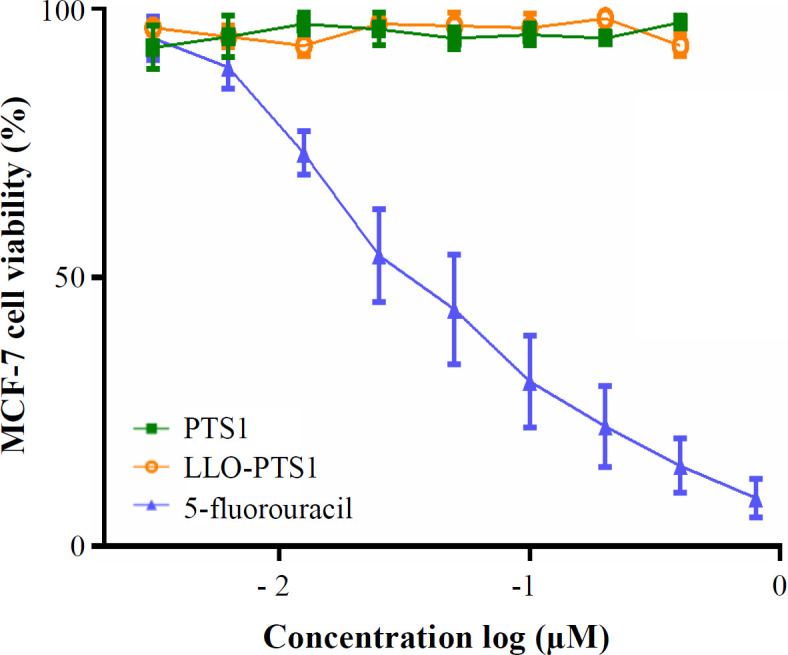
MCF-7 cell viability in the presence of LLO-PTS1. MCF-7 breast cancer cells were cultured and exposed to increasing concentrations of purified PTS1 and LLO-PTS1 proteins. Independently increasing concentrations of 5-fluorouracil was also tested as the positive control

Serial dilutions of recombinant PTS1 protein and LLO-PTS1 fusion protein were applied to the cultured MCF-7 cells in RPMI 1640 media supplemented with 10% FBS. As shown in Figure 6, the purified recombinant PTS1 protein was not cytotoxic to MCF-7 cell line. The 5-fluorouracil was also tested as the positive control. 

## Discussion

*E. coli* has become the most common host for the recombinant protein expression. It is used readily and is economical. In recent years, *SHuffle T7* strain of *E. coli* has been introduced for the cytoplasmic expression of recombinant proteins having disulfide bonds. It benefits from two main features: oxidative environment of cytoplasm and the presence of DsbC chaperone in the cytoplasm^[^^23^^]^. A few recombinant proteins have successfully been produced in *SHuffle* *T7 *cytoplasm^[^^24^^,^^25^^]^. In the present study, recombinant PTS1 and its fusion form, LLO-PTS1, were produced in the *SHuffle* strain. Because the proteins produced in the present study were dialyzed in a buffer free of any oxidant and reducing agent after elution from the affinity column, it seems that the more oxidant environment of *SHuffle T7* cytoplasm can be responsible for the higher efficiency of disulfide bond formation in the protein obtained from this strain.

Chemically inactivated toxoid vaccines possess several adverse effects, thereby demanding for the use of alternative strategies to improve safety. Efficacy of the aP vaccines in generating a Th2, instead of Th1, immune response against pertussis antigens is also the matter of controversy^[^^26^^-^^29^^]^. Subunit vaccines are attracting interest due to the lack of native toxin problems. Using new subunit vaccines has been discussed elsewhere^[^^30^^]^. Higher stability of the subunit vaccines in the formulations prepared for use in populations in comparison with the live vaccines or even high-cost acellular vaccines is another advantage of these substances^[^^31^^]^. 

Lee and colleagues^[^^32^^]^ have found that the fusion of the S1, S3, and filamentous haemagglutinin can be expressed as a single recombinant protein. In addition, the produced recombinant antigen was immunogenic in mice, but the antibody titers against the pertussis toxin subunits showed to be weak. In another study,recombinant *Mycobacterium bovis* BCG was utilized as a host for the production of PTS1 protein. This mode of administration provoked a good cellular immunity in a mouse model, but the level of humoral immunity against the pertussis toxin antigen, as the main virulence factor of pertussis, was low^[^^33^^]^. Diphtheria, tetanus, and pertussis toxin molecule subunits have already been expressed in tobacco and carrot in a soluble form. It has been indicated that these plant-derived proteins could elicit a specific antibody response in animal models^[^^34^^]^. Although there are several advantages in large-scale protein production in plants over the conventional biofactories like the animal cell lines or yeast and bacteria cells, there are yet some challenges in the industrial extraction and purification steps. Recombinant pertussis toxin subunits have also been expressed and purified separately to form oligomers *in vitro*. They bear the potential to form *in vitro* assemblies, which are antigenic, and the antibodies they provoke have been displayed to have inhibitory activities against pertussis toxin^[^^35^^]^. 

Herein, we have produced a new form of genetically inactivated PTS1 using a cloning strategy, which successfully produced two forms of inactivated PTS1 toxin (PTS1 and fusion LLO-PTS1 proteins) in *E. coli* *BL21 DE3* and *SHuffle* T7 strains. However, the rate of the disulfide bond in* SHuffle* T7 strain showed to be more efficient. The analysis of the safety of the proteins produced against a cell line model revealed that both recombinant proteins are safe and non-toxic for the human cell culture model. Our results demonstrated that the recombinant proteins PTS1 and LLO-PTS1 can be expressed in *SHuffle T7* strain cytoplasm and purified in a soluble form with disulfide bonds with high efficiency. Overall, the LLO-PTS1 fusion protein can be formulated as a new generation of the adjuvants to be used as a novel immune-stimulator in the next research studies of pertussis disease. 

## References

[1] Warfel JM, Zimmerman LI, Merkel TJ (2014). Acellular pertussis vaccines protect against disease but fail to prevent infection and transmission in a nonhuman primate model. Proceedings of the national academy of sciences of the United States of America.

[2] Black RE, Cousens S, Johnson HL, Lawn JE, Rudan I, Bassani DG (2010). Child Health Epidemiology Reference Group of WHO and UNICEF Global regional and national causes of child mortality in 2008: a systematic analysis. Lancet.

[3] Trollfors B, Taranger J, Lagergård T, Lind L, Sundh V, Zackrisson G, Lowe CU, Blackwelder W, Robbins JB (1995). A placebo-controlled trial of a pertussis-toxoid vaccine. The new England journal of medicine.

[4] Patterson J, Kagina BM, Gold M, Hussey GD, Muloiwa R (2017). Adverse events following primary and secondary immunisation with whole-cell pertussis: a systematic review protocol. BMJ open.

[5] Poland GA (2012). Pertussis outbreaks and pertussis vaccines: new insights, new concerns, new recommendations?. Vaccine.

[6] Hong JY (2010). Update on pertussis and pertussis immunization. Korean journal of pediatrics.

[7] de Greeff SC, Mooi FR, Westerhof A, Verbakel JMM, Peeters MF, Heuvelman CJ, Elvers LH, Schellekens JFP, de Melker HE (2010). Pertussis disease burden in the household: how to protect young infants. Clinical infectious diseases.

[8] Pittman M (1979). Pertussis toxin: the cause of the harmful effects and prolonged immunity of whooping cough A hypothesis. Reviews of infectious diseases.

[9] Robbins JB, Schneerson R, Keith JM, Miller MA, Kubler-Kielb J, Trollfors B (2009). Pertussis vaccine: a critique. Pediatric infectious disease journal.

[10] Shahin RD, Brennan MJ, Li ZM, Meade BD, Manclark CR (1990). Characterization of the protective capacity and immunogenicity of the 69-kd outer membrane protein of Bordetella pertussis. Journal of experimental medicine.

[11] Coutte L, Locht C (2015). Investigating pertussis toxin and its impact on vaccination. Future microbiology.

[12] Kapil P, Merkel TJ (2019). Pertussis vaccines and protective immunity. Current opinion in immunology.

[13] Reed SG, Orr MT, Fox CB (2013). Key roles of adjuvants in modern vaccines. Nature medicine.

[14] Wallecha A, French C, Petit R, Singh R, Amin A, Rothman J (2012). Lm-LLO-based immunotherapies and HPV-associated disease. Journal of oncology.

[15] Shahabi V, Seavey MM, Maciag PC, Rivera S, Wallecha A (2011). Development of a live and highly attenuated Listeria monocytogenes-based vaccine for the treatment of Her2/neu-overexpressing cancers in human. Cancer gene therapy.

[16] Kumar TD, Balakrishna K, Murali HS, Batra HV (2009). Construction of a recombinant intergenus multidomain chimeric protein for simultaneous expression of haemolysin BL of Bacillus cereus, listeriolysin O of Listeria monocytogenes and enterotoxin B of Staphylococcus aureus. Journal of medical microbiology.

[17] Kim SH, Castro F, Paterson Y, Garvekamp C (2009). High efficacy of a Listeria-based vaccine against metastatic breast cancer reveals a dual mode of action. Cancer research.

[18] Peng X, Treml J, Paterson Y (2007). Adjuvant properties of listeriolysin O protein in a DNA vaccination strategy. Cancer immunology immunotherapy.

[19] Thangudu RR, Vinayagam A, Pugalenthi G, Manonmani A, Offmann B, Sowdhamini R (2005). Native and modeled disulfide bonds in proteins: knowledge-based approaches toward structure prediction of disulfide-rich polypeptides. Proteins.

[20] Dombkowski AA (2003). Disulfide by design: a computational method for the rational design of disulfide bonds in proteins. Bioinformatics.

[21] Krieger E, Koraimann G, Vriend G (2002). Increasing the precision of comparative models with yasara nova-a self-parameterizing force field. Structure, function and genetics.

[22] Jaliani HZ, Farajnia S, Mohammadi SA, Barzegar A, Talebi S (2013). Engineering and kinetic stabilization of the therapeutic enzyme Anabeana variabilis phenylalanine ammonia lyase. Biotechnology and applied biochemistry.

[23] Lobstein J, Emrich CA, Jeans C, Faulkner M, Riggs P, Berkmen M (2012). SHuffle, a novel Escherichia coli protein expression strain capable of correctly folding disulfide bonded proteins in its cytoplasm. Microbial cell factories.

[24] Kong B, Guo GL (2014). Soluble expression of disulfide bond containing proteins FGF15 and FGF19 in the cytoplasm of Escherichia coli. PloS one.

[25] Sermadiras I, Revell J, Linley JE, Sandercock A, Ravn P (2013). Recombinant expression and in vitro characterisation of active Huwentoxin-IV. PloS one.

[26] Robbins JB, Schneerson R, Kubler-Kielb J, Trolifors B, Vinogradoy E, Shiloach J (2014). Toward a new vaccine for pertussis. Proceedings of the national academy of sciences of USA.

[27] Robbins JB, Schneerson R, Keith JM, Shilooach J, Miller M, Trollors B (2007). The rise in pertussis cases urges replacement of chemically-inactivated with genetically-inactivated toxoid for DTP. Vaccine.

[28] Rowe J, Yerkovich ST, Richmond P, Suriyaarachchi D, Fisher E, Feddema L, Loh R, Sly PD, Holt PG (2005). Th2-associated local reactions to the acellular diphtheria-tetanus-pertussis vaccine in 4- to 6-year-old children. Infections immunity.

[29] Locht C (2016). Pertussis: Where did we go wrong and what can we do about it?. Journal of infection.

[30] Robbins JB, Schneerson R, Trollfors B, Sato H, Sato Y, Rappuoli R, Keith JM (2005). The diphtheria and pertussis components of diphtheria-tetanus toxoids-pertussis vaccine should be genetically inactivated mutant toxins. Journal of infection.

[31] Baxter D (2007). Active and passive immunity, vaccine types, excipients and licensing. Occupational medicine(Lond).

[32] Lee SF, Halperin SA, Knight JB, Tait A (2002). Purification and immunogenicity of a recombinant Bordetella pertussis S1S3FHA fusion protein expressed by Streptococcus gordonii. Applied and environmental microbiology.

[33] Nascimento IP, Dias WO, Mazzantini RP, Miyaji EN, Gamberini M, Quintilio W, Gebara VC, Cardoso DF, Paulo LH, Raw I, Winter N, Gicquel B, Rappuoli R, Leite LCC (2000). Recombinant mycobacterium bovis bcg expressing pertussis toxin subunit s1 induces protection against an intracerebral challenge with live Bordetella pertussis in mice. Infection and immunity.

[34] Brodzik R, Spitsin S, Pogrebnyak N, Bandurska K, Portocarrero C, Andryszak K, Koprowski H, Golovkin M (2009). Generation of plant-derived recombinant DTP subunit vaccine. Vaccine.

[35] Burnette WN, Arciniega JL, Mar VL, Burns DL (1992). Properties of pertussis toxin B oligomer assembled in vitro from recombinant polypeptides produced by Escherichia coli. Infection and immunity.

